# Nomograms for Predicting Coexisting Cardiovascular Disease and Prognosis in Chronic Obstructive Pulmonary Disease: A Study Based on NHANES Data

**DOI:** 10.1155/2022/5618376

**Published:** 2022-06-09

**Authors:** Yuanjie Qiu, Yan Wang, Nirui Shen, Qingting Wang, Limin Chai, Jian Wang, Qianqian Zhang, Yuqian Chen, Jin Liu, Danyang Li, Huan Chen, Manxiang Li

**Affiliations:** Department of Respiratory and Critical Care Medicine, The First Affiliated Hospital of Xi'an JiaoTong University, Xi'an 710061, Shaanxi, China

## Abstract

**Background:**

Chronic obstructive pulmonary disease (COPD) is a common chronic disease. Progression is further exacerbated by the coexistence of cardiovascular disease (CVD). We aim to construct a diagnostic nomogram for predicting the risk of coexisting CVD and a prognostic nomogram for predicting long-term survival in COPD.

**Methods:**

The 540 eligible participants selected from the NHANES 2005–2010 were included in this study. Logistic regression analysis was used to construct a diagnostic nomogram for the diagnosis of coexisting CVD in COPD. Cox regression analyses were used to construct a prognostic nomogram for COPD. A risk stratification system was developed based on the total score generated from the prognostic nomogram. We used C-index and ROC curves to evaluate the discriminant ability of the newly built nomograms. The models were also validated utilizing calibration curves. Survival curves were made using the Kaplan–Meier method and compared by the Log-rank test.

**Results:**

Logistic regression analysis showed that gender, age, neutrophil, RDW, LDH, and HbA1c were independent predictors of coexisting CVD and were included in the diagnostic model. Cox regression analysis indicated that CVD, gender, age, BMI, RDW, albumin, LDH, creatinine, and NLR were independent predictors of COPD prognosis and were incorporated into the prognostic model. The C-index and ROC curves revealed the good discrimination abilities of the models. And the calibration curves implied that the predicted values by the nomograms were in good agreement with the actual observed values. In addition, we found that coexisting with CVD had a worse prognosis compared to those without CVD, and the prognosis of the low-risk group was better than that of the high-risk group in COPD.

**Conclusions:**

The nomograms we developed can help clinicians and patients to identify COPD coexisting CVD early and predict the 5-year and 10-year survival rates of COPD patients, which has some clinical practical values.

## 1. Introduction

Chronic obstructive pulmonary disease (COPD) is a common chronic disease that is preventable and treatable, characterized by persistent respiratory symptoms and irreversible airflow limitation [1]. COPD is closely related to chronic bronchitis and emphysema, and they are the most common diseases causing COPD, and most people have some characteristics of both diseases [2]. Epidemiological statistics show that in 2015, an estimated 299 million people worldwide were living with COPD, and more than 3 million people died from this chronic disease [3]. COPD poses a substantial economic burden on society and threatens people's physical and mental health [4].

In recent years, comorbidity has been considered a global problem, which is defined as the coexistence of two or more chronic diseases, and COPD is also a systemic disease, usually associated with other chronic diseases, including cardiovascular disease (CVD), diabetes, lung cancer, osteoporosis, and depression [5–7]. CVD is a common and important comorbidity of COPD, which is associated with worse quality of life and increased all-cause mortality, subsequently increasing the disease burden and economic burden of COPD patients [8–11]. A meta-analysis suggests that patients with COPD are more likely to be diagnosed with CVD than non-COPD populations [12]. CVD and COPD share common risk factors and similar clinical manifestations [13, 14]. Some patients have insufficient understanding of COPD complicated with CVD and easily ignore the diagnosis of COPD combined with CVD. Therefore, it is particularly critical to developing a diagnostic model of COPD coexisting with CVD.

National Health and Nutrition Examination Survey (NHANES) is a cross-sectional survey of the health and nutrition status of the general population of the United States conducted by the National Center for Health Statistics (NCHS) at the Centers for Disease Control and Prevention (CDC). The NHANES program randomly selects participants through a complex multistage cluster sampling probability design, surveys people who do not repeat each year, and each survey includes interviews and physical examinations, involving general demographic data, dietary nutritional status, history of chronic diseases (chronic bronchitis, emphysema, cardiovascular disease, diabetes, etc.), laboratory data, and various health-related problems. NCHS also links data from various surveys to death certificate records from the National Death Index (NDI), providing an opportunity to conduct several studies aimed at investigating the relationship between various health factors and mortality.

To identify specific COPD patients complicated with CVD and low survival rate patients, further developing personalized treatment strategies, improving clinical treatment outcomes, and enhancing the quality of life, this study aimed to use demographic parameters and common hematological parameters from NHANES to construct a diagnostic model for predicting the risk of COPD coexisting CVD and a prognostic model for predicting the prognosis of COPD.

## 2. Methods

### 2.1. Data Sources

Data were obtained from the NHANES database (2005–2010), which contained demographic information, health-related, and healthcare-related characteristics, collected through household interviews and standardized physical examinations at the mobile examination center (MEC) laboratory. NCHS data associated with NDI mortality files updated to 31 December 2015. This research was a retrospective study. All data are available and free to download at https://www.cdc.gov/nchs/nhanes/index.htm and https://www.cdc.gov/nchs/data-linkage/mortality.htm. Data was publicly available, so approval of the Institutional Review Board was not required.

### 2.2. Research Population

The identification of study subjects was confirmed based on household interview questionnaires. Survey participants who had been told by a doctor or other health care provider (HCP) to have emphysema (MCQ160G) and/or chronic bronchitis (MCQ160K) were considered COPD. Survey participants who had been told by a doctor or other HCP to have congestive heart failure (MCQ160B) and/or coronary heart disease (MCQ160C) and/or angina (MCQ160D) and/or heart attack (also known as myocardial infarction) (MCQ160E) were considered CVD. Participants in all included studies were older than 40 years. People who had been told by a doctor or other HCP that they had cancer or any type of malignancy (MCQ220) were excluded from this study. Participants with flu, pneumonia, or ear infections that started during those 30 days (HSQ520) or a stomach or intestinal illness with vomiting or diarrhea that started during those 30 days (HSQ510) were not included in this study. Those with incomplete information on study variables also would be excluded from this study.

### 2.3. Research Variables

The study was followed up until December 31, 2015. Survival time was defined as the time from the date of the NHANES interview to the death of the survey participants (before December 31, 2015) or the end of follow-up. Demographic and clinical information was as follows: age, gender, body mass index (BMI), smoking status (never-smoker, ex-smoker, current-smoker), systolic blood pressure (SBP), diastolic blood pressure (DBP), and comorbid CVD. Hematology indicators included blood cell count (leukocyte, lymphocyte, monocytes, neutrophils, eosinophils, basophils, erythrocyte, hemoglobin, hematocrit (HCT), RDW, MPV), c-reactive protein (CRP), biochemical indicators (albumin, ALT, AST, ALP, BUN, LDH, UA, creatinine, GTT), osmolality, glycosylated hemoglobin (HbA1c), and high-density lipoprotein (HDL). We also investigated the correlation of coexisting CVD in COPD and composite inflammatory markers, including neutrophil-to-lymphocyte ratio (NLR), lymphocyte-to-monocyte Ratio (LMR), and systemic inflammatory response index (SIRI, which is calculated as monocyte count multiplied by neutrophil count divided by lymphocyte count).

### 2.4. Construction of Diagnostic and Prognostic Models

We screened out the factors used to construct a diagnostic model for diagnosing COPD coexisting CVD utilizing stepwise backward logistic regression analysis and formed a visual nomogram. The factors affecting COPD prognosis were screened by performing univariate and multivariate COX regression analysis, and the above-mentioned factors were used to construct a prognostic nomogram of COPD. The accuracy of the model was evaluated with the concordance index (C-index) and receiver operating characteristic (ROC) curve and area under the curve (AUC), and the closer the value was to 1, the higher the accuracy. Use the calibration curve to assess the predictive power of the model. The predictions of a well-calibrated model will fall on the 45-degree diagonal.

### 2.5. Survival Analysis and Construction of Risk Stratification System

The survival curves of the two groups (COPD + CVD group and COPD group) were drawn using the Kaplan–Meier method, and the difference in survival between the two groups was compared using the log-rank test. A risk stratification system was developed based on the total score generated from the prognostic nomogram for each patient. According to the established risk stratification system, those with a risk score higher than the median risk score were assigned to the high-risk group, and those with a lower risk score were assigned to the low-risk group. Kaplan–Meier survival curve was drawn and the log-rank test was used to compare the survival differences between different risk groups.

### 2.6. Statistical Analysis

Depending on the distribution of the data, continuous variables were expressed as mean and standard deviation (SD) or median and interquartile range (IQR). Variables were compared using the two-sample *t*-test or the Wilcoxon rank-sum test with continuity correction based on data normality and homogeneity of variance. Categorical data were presented as absolute values and percentages, and Pearson's chi-square test was used to compare the two groups of categorical variables. Data were organized using Excel, and RStudio version 4.1.2 was used for data analysis. Packages including “readxl,” “car,” “autoReg,” “dplyr,” “officer,” “foreign,” “moonBook,” “rrtable,” “survival,” “survivalROC,” “survminer,” “rms,” “foreign,” and “tableone” were used in *R* software. Statistical significance was set at *p* < 0.05.

## 3. Results

### 3.1. NHANES Database Search

**Figure 1:**
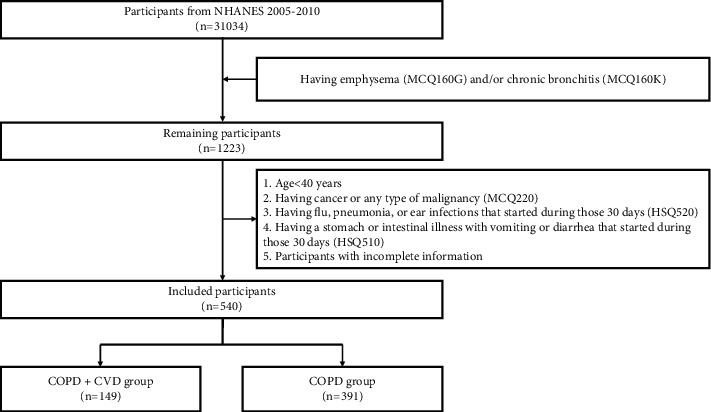
Flow chart of the study protocol. The COPD + CVD group included participants with COPD coexisting with CVD. The COPD group included participants with COPD but without CVD.

31,034 participants were identified from the NHANES database (2005–2010). Through household interviews, 1,223 participants were considered to have COPD. After reviewing the inclusion and exclusion criteria, 540 people were finally confirmed to meet the conditions for further analysis, of which 149 were in the COPD + CVD group, and 391 in the COPD group (participants with COPD but without CVD were included in the COPD group) (Figure 1).

### 3.2. Demographic and Hematological Indicators

Baseline demographic data for both groups were shown in Table 1. Compared with the COPD group, the proportion of male participants was higher in the COPD + CVD group (65.1% vs. 39.6%). The age and BMI of the COPD + CVD group were significantly higher than those of the COPD group. There were no significant differences in blood pressure and smoking status between the two groups.

The hematological indexes of the two groups were shown in Table 2. The leukocyte, neutrophils, RDW, BUN, LDH, UA, creatinine, GTT, osmolality, HbA1c, NLR, and SIRI in the COPD + CVD group were significantly higher than those in the COPD group. However, HDL was lower in the COPD + CVD group. Lymphocyte, monocyte, eosinophil, basophil, erythrocyte, hemoglobin, HCT, MPV, CRP, albumin, ALT, AST, ALP, and LMR were not significantly different.

### 3.3. Diagnostic Model and Nomogram

**Figure 2:**
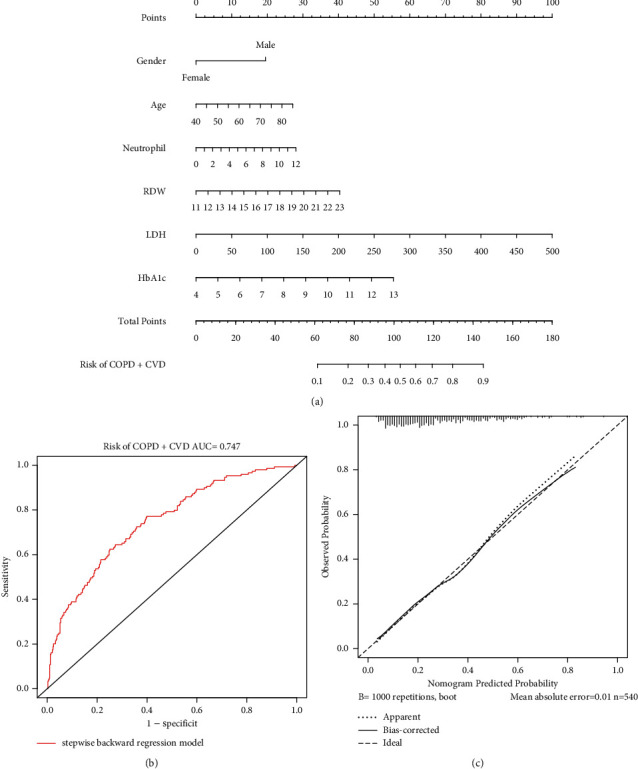
(a) Nomogram showing results of the diagnostic model using gender, age, neutrophil, RDW, LDH, and HbA1c. Each diagnostic factor corresponded to a score, and the total score of an individual patient was obtained by adding the scores of the individual factors, and a straight line was drawn on the axis of the total score to predict the risk of coexisting cardiovascular disease in COPD. (b) The ROC curves for the diagnostic nomogram. (c) The calibration curves of the diagnostic nomogram.

Logistic regression analysis was performed on variables with significant differences between the two groups (gender, age, BMI, leukocyte, neutrophils, RDW, BUN, LDH, UA, creatinine, GTT, osmolality, HbA1c, HDL, NLR, and SIRI). After stepwise backward regression analysis, the following was finally determined: gender (OR 0.36, 95% CI 0.23–0.56, *P* < 0.001), age (OR 1.04, 95% CI 1.02–1.06, *P* < 0.001), neutrophils (OR 1.25, 95% CI 1.04–1.49, *P*=0.015), RDW (OR 1.20, 95% CI 1.03–1.40, *P*=0.022), LDH (OR 1.01, 95% CI. 1.00–1.02, *P*=0.003), and HbA1c (OR 1.34, 95% CI 1.12–1.62, *P*=0.002) were included in the construction of a diagnostic model for diagnosing COPD coexisting with CVD (Table 3), and a visual nomogram was formed (Figure 2(a)). The C-index for this diagnostic model was 0.747. The AUC was also 0.747 (Figure 2(b)). Calibration plots indicated that predictions of the nomogram for the risk of CVD in COPD were highly consistent with actual observations (Figure 2(c)).

### 3.4. Survival Analysis

**Figure 3:**
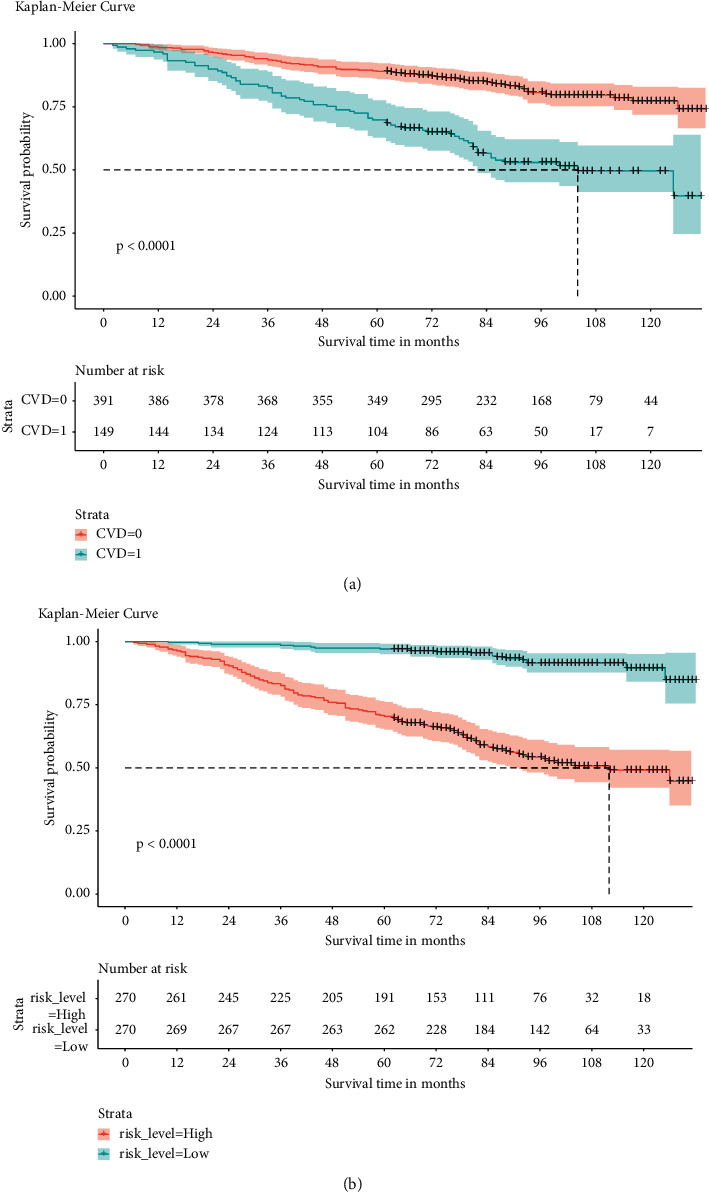
(a) Kaplan–Meier survival curves of CVD group in COPD. CVD = 0 means participants with COPD but without CVD, while CVD = 1 means participants with COPD coexisting with CVD. (b) Kaplan–Meier survival curves in high- and low-risk groups.

The median follow-up for the entire included population was 88 (68–103) months. The median follow-up time for participants in the COPD and COPD + CVD groups was 91(72–104) and 79(51–100) months, respectively. Kaplan–Meier analysis found a significant difference in all-cause mortality between the two groups. Compared with the COPD group, the survival rate of the COPD + CVD group was significantly lower (*P* < 0.0001) (Figure 3(a)).

### 3.5. Prognostic Model and Nomogram

**Figure 4:**
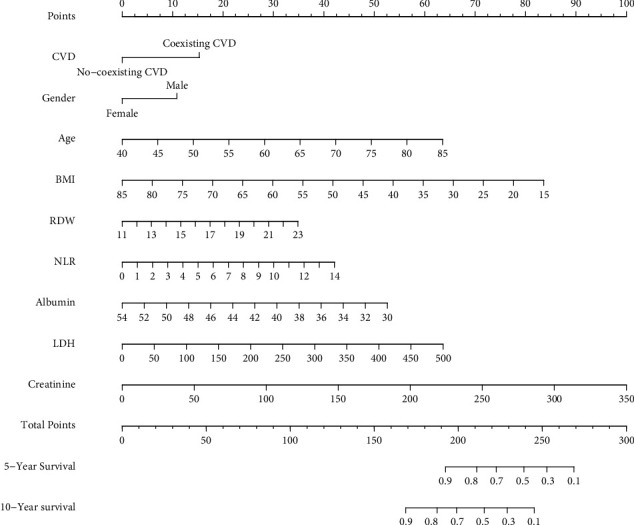
Nomogram showing results of the prognostic model using CVD, gender, age, BMI, RDW, NLR, albumin, LDH, and creatinine for prediction of 5-year, and 10-year survival of COPD. Each prognostic factor corresponded to a score, and the total score of an individual patient was obtained by adding the scores of the individual factors, and a straight line was drawn on the axis of the total score to predict the 5- and 10-year survival of COPD.

**Figure 5:**
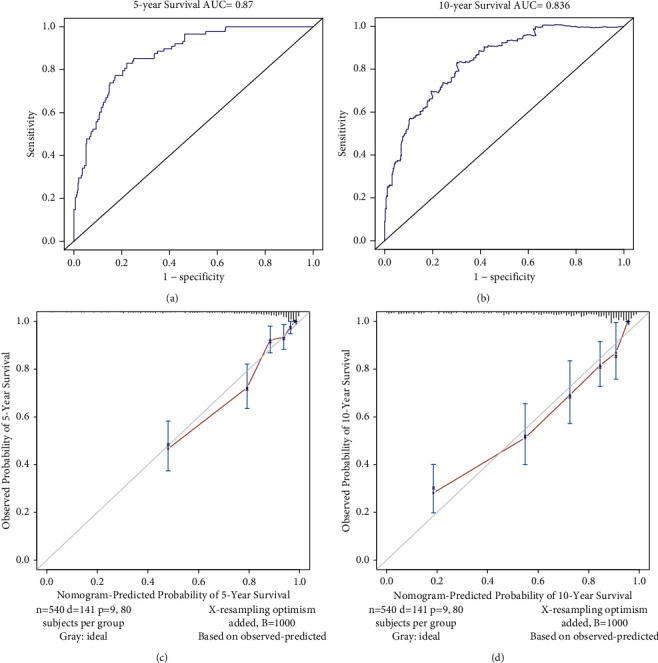
The ROC curves of the prognostic nomogram for predicting the 5-year (a), and 10-year survival (b) in COPD. The calibration curves of the prognostic nomogram for predicting 5-year (c) and 10-year survival (d) in COPD.

We performed univariate and multivariate COX regression analysis to screen factors affecting COPD prognosis. As shown in Table 4, univariate COX regression analysis indicated CVD (*P* < 0.001), gender (*P* < 0.001), age (*P* < 0.001), SBP (*P*=0.001), BMI(*P*=0.038), monocyte (*P*=0.001), erythrocyte (*P*=0.027), RDW (*P* < 0.001), CRP (*P*=0.037), albumin (*P*=0.001), ALP (*P*=0.005), BUN (*P* < 0.001), LDH (*P* < 0.001), UA (*P* < 0.001), creatinine (*P* < 0.001), osmolality (*P* < 0.001), HbA1c (*P*=0.027), HDL (*P*=0.021), NLR (*P* < 0.001), LMR (*P* < 0.001), and SIRI (*P* < 0.001) affected the prognosis of COPD. Further incorporating these factors into multivariate COX regression analysis showed that CVD (HR 1.68, 95% CI 1.16–2.44, *P*=0.006), gender (HR 0.56, 95% CI 0.36–0.88, *P*=0.011), age (HR 1.06, 95% CI 1.04–1.08, *P* < 0.001), BMI (HR 0.95, 95% CI 0.92–0.98, *P*=0.002), RDW (HR 1.00, 95% CI 1.00–1.25, *P*=0.046), albumin (HR 0.92, 95% CI 0.87–0.97, *P*=0.002), LDH (HR 1.01, 95% CI 1.00–1.01, *P*=0.022), creatinine (HR 1.01, 95% CI 1.00–1.02, *P*=0.023), and NLR (HR 1.35, 95% CI 1.06–1.71, *P*=0.014) were independent prognostic factors for COPD. The selected factors above were used to construct a prognostic nomogram of COPD (Figure 4). The C-index for this prognostic model was 0.819. The AUC of this prognostic nomogram for predicting 5- and 10-year survival was 0.870 and 0.836, respectively (Figures 5(a), 5(b)). The calibration curve showed that the 5- and 10-year survival predicted by the nomogram was in good agreement with the actual observations (Figures 5(c), 5(d)).

### 3.6. Risk Stratification System

According to the established risk stratification system, there were 270 participants in both high- and low-risk groups. We then plotted the survival Kaplan–Meier curves for each low- and high-risk group (Figure 3(b)). In this system, we found that the low-risk group had a better prognosis than the high-risk group (*P* < 0.0001).

## 4. Discussion

In this study, we used data from the NHANES database to construct practical nomograms to predict the risk of CVD in COPD and to predict the 5-year and 10-year survival of COPD based on easily available demographic information, clinical data, and common hematological parameters. The results showed that the AUC in the model predicting the risk of CVD in COPD was 0.747, and the AUC in the model predicting the 5-year and 10-year survival of COPD was 0.870 and 0.836, respectively. Male gender, advanced age, neutrophilia, increased RDW, high LDH, and high HbA1c were independent predictors of COPD coexisting CVD. CVD, male gender, advanced age, low BMI, increased RDW, low serum albumin, high LDH, high serum creatinine, and high NLR were independent predictors of COPD prognosis.

As a common and important comorbidity of COPD, the presence of CVD is associated with exacerbations and increased mortality [8]. Our univariate Cox regression analysis showed that coexisting CVD (HR 3.05, 95% CI 2.19–4.25, *P* < 0.001) affected the prognosis of COPD patients, and multivariate Cox regression analysis further showed that coexisting CVD (HR 1.68, 95% CI 1.16–2.44, *P*=0.006) was an independent prognostic factor for COPD. The study by Ulf Nilsson et al. also showed that in patients with COPD, elevated hs-cTnI both independently (HR 2.72, 95% CI 1.46–5.07) and in combination with ischemic ECG abnormalities (HR 4.54, 95% CI 2.25–9.13) were associated with an increased risk of mortality [15]. To reduce the risk of mortality in patients with COPD, effective identification of CVD was needed. Elevated LDH levels may be associated with CVD risk [16]. Our study showed that LDH levels were significantly higher in the COPD + CVD group than in the COPD group (145.00U/L vs. 134.00U/L, *P* < 0.001), and multivariate Cox regression analysis indicated that a high level of LDH (HR 1.01, 95% CI 1.00–1.01, *P*=0.022) was an independent predictor of COPD prognosis.

It is well known that demographic factors affect the occurrence and development of diseases. A study by Elizabeth RC Millett et al. found that the incidence of myocardial infarction in men was higher than in women, with an incidence rate of 24.35 (95% CI 23.57–25.16) and 7.76 (95% CI 7.37–8.16) per 10,000 person-years, respectively [17]. In our study, multivariate logistic regression analysis showed that the female gender (OR 0.41, 95% CI 0.24–0.67, *P* < 0.001) was a protective factor for cardiovascular disease, which was consistent with the results of Elizabeth Millett et al. We suspected that the reason may be related to the levels of specific hormones in women, such as estrogen, which has a certain degree of cardiovascular protection [18]. Age is also an important risk factor for many chronic diseases, including COPD and CVD [6]. We found that among COPD patients, older age was more likely to have CVD, which was consistent with a previous survey conducted in China [19]. Results of a prospective study conducted by Ernesto Crisafulli et al. suggested that age was a determinant of death in hospitalized patients with AECOPD [20]. Our multivariate COX regression analysis also indicated that age (HR 1.06, 95% CI 1.04–1.08, *P* < 0.001) was an independent prognostic factor for COPD. Demographic characteristics such as gender and age should be considered when developing strategies for the management of COPD and its comorbidities.

As we all know, smoking is one of the risk factors for COPD and CVD [6]. However, our study found that smoking status was not significantly different between the COPD + CVD group and the COPD group (*P*=0.121), which may be precise because both groups of people were more prone to smoking. Our study also did not find that smoking significantly affected the long-term prognosis of COPD (*P*=0.108), which was consistent with the results of two previous studies [21, 22]. Further studies are needed on the effect of smoking on the risk of CVD and the prognosis of COPD.

Systemic inflammatory mechanisms are important for the pathogenesis of COPD and CVD [5]. Wang et al. performed a prospective study. The results suggested that high neutrophil was linked to higher CVD risk [23]. The study by Guler Ozgul et al. showed that the presence of CVD (OR 4.3, 95% CI 1.3–11, *P*=0.01) in COPD was independently associated with elevated RDW [24]. Panagiotis Paliogiannis et al. showed that NLR was a valuable predictor of mortality in COPD by reviewing multiple studies [25]. A study by Ekrem Cengiz Seyhan et al. showed that RDW level [HR 1.12, 95% CI (1.01–1.24), *P*=0.01] was independently associated with mortality in stable COPD patients [26]. Our findings were consistent with these previous findings. In our study, the levels of neutrophils and RDW in participants were higher in the COPD + CVD group than in the COPD group. Multivariate Cox regression analysis indicated that high levels of NLR and RDW were independent predictors of long-term survival in COPD participants. Higher neutrophils, NLR, and RDW levels may reflect underlying chronic inflammation, which may contribute to increased CVD risk and increased COPD mortality [25, 27, 28].

Patients with COPD could experience a state of malnutrition, and malnutrition was associated with increased mortality [29]. BMI was the clinically important and simplest assessment of nutritional status, and a BMI <18.5 kg/m^2^ was considered malnutrition [30]. Albumin was a routine hematological indicator of malnutrition biomarker, and patients were considered malnourished when their serum albumin <35 g/L [31]. Previous studies have demonstrated that low BMI and low serum albumin levels were significant independent predictors of increased long-term mortality [21, 22, 32, 33]. Our present study also showed that low BMI and low serum albumin levels were associated with a worse prognosis in COPD patients. The mechanism of their poor long-term prognosis may be further respiratory muscle weakness and decreased immune response due to poor nutritional status. Therefore, the nutritional status of patients with COPD should be properly evaluated. Early nutritional intervention could improve the prognosis of patients.

In recent years, the relationship between HbA1c and the risk of CVD has attracted increasing attention [34–38]. A meta-analysis of HbA1c involving nine studies and yielding 49,099 participants, found a close relationship between HbA1c levels and coronary heart disease risk [34]. In a large prospective cohort study with type 2 diabetes, high levels of HbA1c were associated with CVD (HR 1.08, 95%CI 1.06–1.10) and MI (HR 1.08, 95%CI 1.04–1.11), confirming HbA1c Elevation was an independent predictor of CVD [35]. A meta-analysis by Elizabeth Selvin et al. involving patients with type 1 diabetes (*n* = 1688) and type 2 diabetes (*n* = 7435) showed a combined relative risk of CVD of 1.18 [36]. Recently, de Jong et al. conducted a prospective cohort study and found that every 1% increase in HbA1c independent of diabetes status led to an 18% increased risk of MI [37]. The association between HbA1c and common carotid artery intima-media thickness (CCA-IMT) may contribute to a key link between high HbA1c levels and CVD in nondiabetic adults [38]. In our study, univariate logistic regression analysis indicated that high HbA1c was a risk factor for CVD in COPD patients (OR 1.42, 95% CI 1.20–1.68, *P* < 0.001), and multivariate logistic regression analysis further revealed that high HbA1c was an independent predictor of CVD (OR 1.29, 95% CI 1.07–1.57, *P*=0.009). Therefore, clinicians should consider that elevated HbA1c was more likely to be associated with CVD during COPD management.

In our study, multivariate COX regression analysis showed that elevated creatinine (HR 1.01, 95% CI 1.00–1.02, *P*=0.023) was associated with poor prognosis in COPD. A previous study conducted in the ICU by He et al. also showed higher serum creatinine levels in COPD patients in the death group, which was consistent with our findings [39]. High creatinine levels indicated poor kidney function. Abnormal renal function in COPD patients may be related to lung and renal endothelial damage, and the mechanism may be explained by increased tissue oxidative stress and levels of advanced glycation end products (AGEs) and receptors for AGEs (RAGE) in lung and renal endothelial cells [40].

Because the occurrence and development of COPD are complex and diverse, the combined application of markers may be the focus of early identification of comorbidities and improvement of long-term prognosis in COPD in the future. Several tools have been developed for the identification of COPD comorbidities and COPD mortality [41–43]. The model developed by Fermont et al. to predict cardiovascular risk included conventional CVD risk factors (age, gender, smoking, HDL, total cholesterol, SBP, diabetes, and hypertension medications) with a C-index of 0.689 and the addition of the 6-minute walk test (6-MWT) improved model discrimination with a C-index of 0.727 [41]. While the C-index of our model for predicting CVD amounted to 0.747, the parameters included (gender, age, peripheral blood neutrophil, RDW, LDH, HbA1c) were easily obtained and the model was presented as a nomogram. Compared with the prediction model developed by Shi et al. [42], the parameters we included in the model were more simplified, and the nomogram was presented in a more intuitive form, which can effectively help clinicians and patients to early identify COPD coexisting CVD. Yukiyo Sakamoto et al. developed a nomogram to predict COPD in-hospital mortality based on variables including age, gender, BMI, disturbance of consciousness, severe dyspnea, history of mechanical ventilation, pneumonia, and comorbid asthma on admission, with a C-index of 0.775 [43]. Compared with his nomogram, we included CVD, gender, age, BMI and peripheral blood RDW, etc., adding hematological indexes, and the C-index for predicting 5-year and 10-year survival were 0.870 and 0.836, respectively, with more accurate predictive ability.

This study had some limitations. First, since the follow-up data was only updated to December 31, 2015, we only included the data from NHANES 2005–2010 to ensure a long enough follow-up time. Second, due to the lack of spirometry data in the NHANES database 2005–2006, the selection of our included study subjects relied on questionnaire data rather than GOLD diagnostic criteria for hospitalized COPD, which may be biased to some extent. However, in a previous study based on the NHANES database 2007–2012 comparing AUC between COPD determined by spirometry data and questionnaire data, there was little difference in AUC which demonstrated a difference between COPD patients identified by the two modalities was small [42]. Third, since there were no data on lung function, we could not assess the relationship between COPD severity with CVD and COPD mortality, and the established nomogram did not include the COPD stage. In addition, the model we constructed only utilized the data of the modeling itself to validate the predictive effect of the model, and external data should be used to further validate the accuracy of the model. To better use nomograms in clinical practice to predict the risk of coexisting CVD in COPD and the long-term prognosis of COPD, it is necessary to carry out more rigorous, multicenter prospective studies in the future to validate our constructed model.

## 5. Conclusion

We developed nomograms to predict the risk of coexisting CVD in COPD and to predict the 5- and 10-year survival of COPD. This nomogram can help clinicians and patients to early identify COPD coexisting with CVD and predict the 5-year and 10-year survival rates of COPD patients, which can provide appropriate clinical information for patients and clinicians, develop personalized treatment strategies, and improve the quality of life.

## Figures and Tables

**Table 1:** Baseline demographic characteristics of the included participants.

Variables		COPD group (*n* = 391)	COPD + CVD group (*n* = 149)	*P*
Follow-up time, months		91.00 [72.00, 104.00]	79.00 [51.00, 100.00]	<0.001

Gender	Male, *n* (%)	155 (39.6)	97 (65.1)	<0.001
	Female, *n* (%)	236 (60.4)	52 (34.9)	

Age, years		61.00 [50.00, 72.00]	67.00 [60.00, 76.00]	<0.001

SBP, mmHg		126.00 [116.00, 142.00]	128.00 [116.00, 144.00]	0.713

DBP, mmHg		70.00 [62.00, 76.00]	70.00 [60.00, 78.00]	0.544

BMI, kg/m^2^		28.52 [24.34, 34.05]	29.90 [26.14, 35.49]	0.033

Smoke	Never-smoker, *n* (%)	116 (29.7)	34 (22.8)	0.121
	Ex-smoker, *n* (%)	148 (37.9)	70 (47.0)	
	Current-smoker, *n* (%)	127 (32.5)	45 (30.2)	

SBP, systolic blood pressure; DBP, diastolic blood pressure; BMI, body mass index; IQR, interquartile range. Values were presented as median [interquartile range (IQR)] or *n* (%).

**Table 2:** Baseline hematological indexes of the included participants.

Variables	COPD group (*n* = 391)	COPD + CVD group (*n* = 149)	*p*
Leukocyte, 10 ^ 9 cells/L	7.10 [5.90, 8.80]	7.50 [6.40, 8.80]	0.041
Lymphocyte, 10 ^ 9 cells/L	2.00 [1.60, 2.50]	2.00 [1.40, 2.60]	0.704
Monocyte, 10 ^ 9 cells/L	0.50 [0.40, 0.70]	0.60 [0.50, 0.70]	0.092
Neutrophil, 10 ^ 9 cells/L	4.20 [3.40, 5.40]	4.60 [3.70, 5.70]	0.013
Eosinophil, 10 ^ 9 cells/L	0.20 [0.10, 0.30]	0.20 [0.10, 0.30]	0.046
Basophil, 10 ^ 9 cells/L	0.00 [0.00, 0.10]	0.00 [0.00, 0.10]	0.081
Erythrocyte, 10 ^ 12 cells/L	4.58 (0.50)	4.56 (0.51)	0.659
Hemoglobin, g/dL	14.10 [13.10, 15.20]	14.10 [12.80, 15.10]	0.671
HCT, %	41.30 (4.43)	41.33 (4.61)	0.944
RDW, %	12.90 [12.40, 13.60]	13.40 [12.70, 14.30]	<0.001
MPV, fL	7.70 [7.30, 8.30]	7.90 [7.30, 8.40]	0.104
CRP, mg/dL	0.23 [0.10, 0.75]	0.30 [0.14, 0.71]	0.109
Albumin, g/L	41.00 [39.00, 44.00]	41.00 [39.00, 43.00]	0.43
ALT, U/L	20.00 [15.00, 26.50]	21.00 [18.00, 28.00]	0.064
AST, U/L	23.00 [20.00, 28.50]	24.00 [21.00, 29.00]	0.225
ALP, U/L	72.00 [59.00, 86.00]	72.00 [60.00, 89.00]	0.523
BUN, mg/dL	13.00 [10.00, 16.00]	14.00 [10.00, 20.00]	0.017
LDH, U/L	134.00 [119.00, 150.00]	145.00 [126.00, 165.00]	<0.001
UA, *µ*mol/L	321.20 [258.75, 380.70]	350.90 [297.40, 428.30]	<0.001
Creatinine, *µ*mol/L	76.91 [63.65, 90.17]	88.40 [72.49, 106.08]	<0.001
GTT, U/L	22.00 [15.00, 35.00]	25.00 [17.00, 41.00]	0.006
Osmolality, mmol/kg	279.00 [275.00, 282.00]	280.00 [276.00, 284.00]	0.009
HbA1c, %	5.60 [5.40, 6.00]	5.90 [5.50, 6.70]	<0.001
HDL, mmol/L	1.34 [1.09, 1.66]	1.16 [1.01, 1.45]	<0.001
NLR	2.12 [1.58, 2.80]	2.28 [1.62, 3.33]	0.042
LMR	3.80 [2.73, 4.75]	3.50 [2.40, 4.67]	0.058
SIRI	1.11 [0.78, 1.69]	1.33 [0.85, 2.03]	0.006

HCT: hematocrit. RDW: red cell distribution width. MPV: mean platelet volume. CRP: C-reactive protein. ALT: alanine aminotransferase. AST: aspartate aminotransferase. ALP: alkaline phosphatase. BUN: blood urea nitrogen. LDH: lactate dehydrogenase. UA: uric acid. GTT: gamma-glutamyl transferase. HbA1c: glycosylated hemoglobin. HDL: high-density lipoprotein. NLR: neutrophil count to lymphocyte count ratio. LMR: lymphocyte count to monocyte count ratio. SIRI: systemic Inflammation Response Index. Values were presented as mean (SD) or median [interquartile range (IQR).

**Table 3:** Stepwise backward logistic regression analysis for the risk of CVD in COPD.

Dependent:	OR (univariable)	OR (multivariable)	OR (final)
Gender (female)	0.35 (0.24–0.52, *p* < 0.001)	0.41 (0.24–0.67, *p* < 0.001)	0.36 (0.23–0.56, *p* < 0.001)
Age, years	1.04 (1.02–1.06, *p* < 0.001)	1.04 (1.02–1.06, *p* < 0.001)	1.04 (1.02–1.06, *p* < 0.001)
BMI, kg/m^2^	1.02 (1.00–1.05, *p*=0.055)	—	—
Leukocyte, 10 ^ 9 cells/L	1.03 (0.97–1.11, *p*=0.339)	—	—
Neutrophil, 10 ^ 9 cells/L	1.14 (1.01–1.28, *p*=0.029)	1.22 (1.02–1.48, *p*=0.034)	1.25 (1.04–1.49, *p*=0.015)
RDW, %	1.31 (1.15–1.51, *p* < 0.001)	1.19 (1.02–1.39, *p*=0.031)	1.20 (1.03–1.40, *p*=0.022)
BUN, mg/dL	1.05 (1.02–1.07, *p* < 0.001)	0.99 (0.95–1.03, *p*=0.692)	—
LDH, U/L	1.01 (1.00–1.02, *p* < 0.001)	1.01 (1.00–1.02, *p*=0.007)	1.01 (1.00–1.02, *p*=0.003)
UA, *µ*mol/L	1.00 (1.00–1.01, *p* < 0.001)	1.00 (1.00–1.00, *p*=0.218)	1.00 (1.00–1.00, *p*=0.075)
Creatinine, *µ*mol/L	1.02 (1.01–1.02, *p* < 0.001)	1.00 (0.99–1.01, *p*=0.572)	—
GTT, U/L	1.00 (1.00–1.01, *p*=0.030)	1.00 (1.00–1.01, *p*=0.236)	—
Osmolality	1.06 (1.02–1.09, *p* < 0.001)	1.02 (0.97–1.06, *p*=0.521)	—
HbA1c, %	1.42 (1.20–1.68, *p* < 0.001)	1.29 (1.07–1.57, *p*=0.009)	1.34 (1.12–1.62, *p*=0.002)
HDL, mmol/L	0.42 (0.25–0.67, *p* < 0.001)	0.76 (0.42–1.34, *p*=0.352)	—
NLR	1.15 (1.01–1.30, *p*=0.034)	1.02 (0.78–1.31, *p*=0.892)	—
SIRI	1.26 (1.04–1.53, *p*=0.017)	0.79 (0.52–1.20, *p*=0.269)	0.78 (0.58–1.05, *p*=0.106)

RDW: red cell distribution width. BUN: blood urea nitrogen. LDH: lactate dehydrogenase. UA: uric acid. GTT: gamma-glutamyl transferase. HbA1c: glycosylated hemoglobin. HDL: high-density lipoprotein. NLR: neutrophil count to lymphocyte count ratio. SIRI: systemic inflammation response index. OR: Odds ratio.

**Table 4:** Univariate and multivariate COX regression analysis for predicting long-term mortality in COPD.

Dependent	HR (univariable)	HR (multivariable)
CVD (coexisting with CVD)	3.05 (2.19–4.25, *p* < 0.001)	1.68 (1.16–2.44, *p*=0.006)
Gender (female)	0.41 (0.29–0.58, *p* < 0.001)	0.56 (0.36–0.88, *p*=0.011)
Age, years	1.08 (1.07–1.10, *p* < 0.001)	1.06 (1.04–1.08, *p* < 0.001)
SBP, mmHg	1.01 (1.01–1.02, *p*=0.001)	1.01 (1.00–1.02, *p*=0.234)
DBP, mmHg	0.99 (0.98–1.00, *p*=0.056)	—
BMI, kg/m^2^	0.98 (0.95–1.00, *p*=0.038)	0.95 (0.92–0.98, *p*=0.002)
Smoke	1.19 (0.96–1.47, *p*=0.108)	—
Leukocyte, 10 ^ 9 cells/L	1.04 (0.99–1.09, *p*=0.150)	—
Lymphocyte, 10 ^ 9 cells/L	1.02 (0.94–1.12, *p*=0.581)	—
Monocyte, 10 ^ 9 cells/L	2.68 (1.46–4.93, *p*=0.001)	1.65 (0.80–3.42, *p*=0.175)
Neutrophil, 10 ^ 9 cells/L	1.05 (0.95–1.17, *p*=0.318)	—
Eosinophil, 10 ^ 9 cells/L	1.93 (0.86–4.32, *p*=0.109)	—
Basophil, 10 ^ 9 cells/L	0.92 (0.04–18.84, *p*=0.955)	—
Erythrocyte, 10 ^ 12 cells/L	0.69 (0.49–0.96, *p*=0.027)	0.97 (0.66–1.43, *p*=0.869)
Hemoglobin, g/dL	0.91 (0.82–1.01, *p*=0.081)	—
HCT, %	0.99 (0.95–1.02, *p*=0.436)	—
RDW, %	1.26 (1.16–1.37, *p* < 0.001)	1.12 (1.00–1.25, *p*=0.046)
MPV, fL	0.99 (0.82–1.19, *p*=0.875)	—
CRP, mg/dL	1.13 (1.01–1.26, *p*=0.037)	0.98 (0.84–1.15, *p*=0.830)
Albumin, g/L	0.92 (0.88–0.97, *p*=0.001)	0.92 (0.87–0.97, *p*=0.002)
ALT, U/L	0.99 (0.97–1.00, *p*=0.082)	—
AST, U/L	1.00 (0.99–1.01, *p*=0.833)	—
ALP, U/L	1.01 (1.00–1.02, *p*=0.005)	1.00 (1.00–1.01, *p*=0.116)
BUN, mg/dL	1.06 (1.04–1.07, *p* < 0.001)	1.02 (0.99–1.06, *p*=0.172)
LDH, U/L	1.01 (1.00–1.01, *p* < 0.001)	1.01 (1.00–1.01, *p*=0.022)
UA, *µ*mol/L	1.00 (1.00–1.00, *p* < 0.001)	1.00 (1.00–1.00, *p*=0.609)
Creatinine, µmol/L	1.01 (1.01–1.02, *p* < 0.001)	1.01 (1.00–1.02, *p*=0.023)
GTT, U/L	1.00 (1.00–1.01, *p*=0.057)	—
Osmolality, mmol/Kg	1.05 (1.02–1.09, *p* < 0.001)	0.97 (0.94–1.01, *p*=0.128)
HbA1c, %	1.15 (1.02–1.30, *p*=0.027)	1.14 (0.96–1.36, *p*=0.127)
HDL, mmol/L	0.62 (0.41–0.93, *p*=0.021)	1.06 (0.66–1.70, *p*=0.820)
NLR	1.29 (1.19–1.40, *p* < 0.001)	1.35 (1.06–1.71, *p*=0.014)
LMR	0.82 (0.73–0.92, *p* < 0.001)	1.08 (0.98–1.20, *p*=0.103)
SIRI	1.44 (1.26–1.64, *p* < 0.001)	0.80 (0.54–1.19, *p*=0.276)

CVD: cardiovascular disease; SBP: systolic blood pressure; DBP: diastolic blood pressure; BMI: body mass index; HCT: hematocrit. RDW: red cell distribution width. MPV: mean platelet volume. CRP: C-reactive protein. ALT: alanine aminotransferase. AST: aspartate aminotransferase. ALP: alkaline phosphatase. BUN: blood urea nitrogen. LDH: lactate dehydrogenase. UA: uric acid. GTT: gamma-glutamyl transferase. HbA1c: glycosylated hemoglobin. HDL: high-density lipoprotein. NLR: neutrophil count to lymphocyte count ratio. LMR: lymphocyte count to monocyte count ratio. SIRI: systemic inflammation response index; HR: hazard ratio.

## Data Availability

All data are available and free to download at https://www.cdc.gov/nchs/nhanes/index.htm and https://www.cdc.gov/nchs/data-linkage/mortality.htm.
